# Polysaccharide Composition of Dietary Fiber During Raspberry and Blackberry Juice Production

**DOI:** 10.3390/molecules30102098

**Published:** 2025-05-09

**Authors:** Monika Kosmala, Joanna Milala, Elżbieta Karlińska

**Affiliations:** Institute of Food Technology and Analysis, Lodz University of Technology, Stefanowskiego Street 2/22, 90-537 Lodz, Poland; joanna.milala@p.lodz.pl (J.M.); elzbieta.karlinska@p.lodz.pl (E.K.)

**Keywords:** *Rubus idaeus* L., *Rubus fruticosus* L., water-binding capacity, oil-holding capacity, swelling

## Abstract

Fiber is one of the most important ingredients of fruit that has an influence on the gastrointestinal tract and biochemical parameters of blood. Fiber has texturizing functions in food processing. The fiber’s properties (water-binding capacity, swelling, and oil-holding capacity) and polysaccharide composition obtained from raspberry and blackberry fruit, juice, and pomace, divided into seed and seedless fractions, were determined. The seedless fraction contains more hemicelluloses and homogalacturonan with higher water-binding capacities, swelling, and oil-holding capacities, and the seeds contain more cellulose, and their physical abilities are much lower. Water-binding capacities were from 2.7 to 14.9 g/g, swelling from 3.3 to 11.1 mL/g, and oil-holding capacities from 8.0 to 16.5 g/g. The sequential extraction of fruit fiber showed that the main fraction was the Residue, followed by the weak alkali extractable pectin (DASP) and the hemicellulose (CASP). Water-extractable pectin (WSP) and chelating-agent extractable pectin (ChSP) both constituted 8–9% of AIS each. In the pomace, the main fraction was the Residue (40% AIS), followed by CASP (16% AIS), DASP and ChSP (6–7% AIS), and WSP and WR (3% AIS). While seeds are composed mostly of Residue (52–57% AIS vs. 24–36% AIS in seedless), the seedless part shares of CASP hemicelluloses were higher (24–28% AIS vs. 12–15% in seeds). In the seedless part, there was also more water-soluble pectin (WSP) (4–5% vs. 2–3% in seeds). Seedless fraction is rich in hemicellulose and has higher water-binding properties and oil-holding capacities compared to seeds, and that is why it could be a source of functional berry polysaccharides.

## 1. Introduction

Raspberries (*Rubus idaeus* L.) and blackberries (*Rubus fruticosus* L.) are fruits that are often eaten fresh or frozen. They are also often processed into jams and juices. Raspberries and blackberries are a source of valuable antioxidant ingredients such as polyphenols and vitamins C, A, and E. They also contain dietary fiber and essential and trace minerals [1,2].

The juice production process produces pomace. Raspberry and blackberry fruit pomace are a source of phenolic compounds [3], with ellagitannins being the most abundant, followed by anthocyanins, flavan-3-ols, and flavonols [4]. Raspberry and blackberry pomace primarily comprises seeds and other parts, such as pulps and peels (seedless). The juice production process produces 10–12% of wet pomace. After drying, dry pomace constitutes 5–7% of the initial raw material (fruit), of which the seed fraction constitutes 92–95% [4]. Raspberry and blackberry pomace is a concentrated source of various fiber fractions demonstrating notable functional properties like water-binding capacity (WBC), oil-holding capacity, and swelling capacity. Moreover, these anthocyanin-rich pomaces are interesting due to their health-promoting properties and intensive color [5]. The functional properties of dietary fiber are associated with the physicochemical characteristics of cell wall polysaccharides, varying according to their composition [6,7]. Fiber with high water retention capacity is able to increase fecal bulk and reduce gastrointestinal transit time. Fiber swelling is also able to increase the viscosity of the digest, which slows the absorption of nutrients from the intestinal mucosa and lowers the postprandial blood glucose and insulin response [8]. The inclusion of fiber with high water-binding capacities in the diet of male rats appeared to facilitate the modulation of the viscosity of stomach digestion and the reduction of food intake [8]. In food technology, dietary fiber with high water retention capacities acts as a functional ingredient able to modify the viscosity and texture of food products like pies, and bread, and fiber with high-fat absorption capacities allows stabilization of fat in emulsion-based products like processed meats, pâtés, and sausage fillings [7,9]. Berry seeds contain oil, which is the source of tocopherols (620.1–2166.7 mg kg^−1^) and α-linolenic acid (above 37%) [10], but the most prevalent part of pomace is dietary fiber, constituting 47–65% of pomace dry matter [11]. Raspberry pomace addition to rats fed a high-fat diet decreased liver cholesterol, hepatic fibroblast growth factor receptor 4, peroxisome proliferator-activated receptor alpha, cecal ammonia, and favorably changed bile acids profile in the cecum [11]. Raspberry pomace increases the production of bacterial short-chain fatty acids (SCFAs), especially acetic and butyric acid, which are important for the health of the colon, as butyrate in the colonic enterocytes is rapidly absorbed and metabolized and acts as an anticancerogenic, whereas acetic acid is converted to acetyl-CoA, contributing to lipogenesis [11,12]. Blackberry fiber, divided into polyphenols incorporated into the rat diet, also increased the production of propionate and butyrate in the cecum and improved the blood lipid profile. While extracted polyphenols beneficially decreased the activity of cecal β-glucuronidase, they may have also increased cholesterol levels in blood [13].

When natural polysaccharides are extracted from edible fruits, they possess various important biological activities themselves, such as antioxidant, immunological, and hypoglycemic [14,15,16,17,18,19]. Dragon fruit pomace-derived polysaccharides in mice fed a high-fat diet were proven to significantly decrease body weight increase, abdominal fat accumulation, total cholesterol, triglycerides, and LDL-C concentrations, as well as an elevation in HDL-C concentrations. Moreover, the dragon fruit polysaccharides improved glucose tolerance and prevented fat accumulation in the liver and adipose tissue. Moreover, dragon fruit polysaccharides exhibited anti-inflammatory properties, evidenced by reduced levels of pro-inflammatory cytokines (TNF-α, IL-1β, and IL-6) in the liver. Also, gut microbiota analysis indicated a shift toward beneficial bacteria (*Romboutsia, Lachnospiraceae*, *Coriobacteriaceae*, and *Blautia*) [20]. Molecular weight, content of arabinose, galactose, or glucuronic acid, and glycosyl linkage patterns of →3)-Arap-(1→, Araf-(1→, and →4)-Galp-(1→ are the main structural factors greatly affecting their properties [17].

Berry polysaccharides have been studied in vitro assays expressing anticancer activities [21], although there still exists a need to evaluate them in vivo studies using suitable animal models. However, the anticancer activity of the polysaccharides has been expressed in different units, making their comparison to each other and conventional anticancer agents difficult. That is why a comprehensive investigation of the structure-activity relationship of the polysaccharides is still needed [22].

The extracted polysaccharides can be used in the food industry. Blackberry polysaccharide was found beneficial for the quality improvement of meat products by the high cross-linking to the myofibrillar protein of chicken breast meat network and stabilizing the water distribution [23]. Traditionally, polysaccharides from berries are obtained by hot water extraction. However, prolonged exposure to high temperatures may lead to the degradation of polysaccharides. Other methods include the use of alkalis or acids, but this may lead to the hydrolysis of polysaccharides and require strict process control. To increase the yield of extraction, advanced and effective extraction methods have been developed, such as microwave-assisted extraction (MAE), ultrasound-assisted extraction (UAE), and enzyme-assisted extraction (EAE). However, prolonged exposure to ultrasonic or microwave radiation may lead to polysaccharide degradation and structural alterations caused by mechanical shear and localized high temperatures [19]. New extraction techniques are also emerging in berry polysaccharide extraction, like supercritical fluid extraction (SFE) [24].

Although there are studies that present the nutritional and polyphenol composition of raspberry and blackberry pomace, sometimes even divided into seed and seedless fractions, proving that they are a good source of dietary fiber and ellagitannins with beneficial physiological responses in the gastrointestinal tract [11,13], few studies are devoted to the polysaccharide composition of dietary fiber of the seed and seedless fractions. The aim of our study was to determine the changes occurring in the qualitative and quantitative composition of dietary fiber during juice production. Which polysaccharides pass from fruit to juice, and which remain in pomace? Does the composition of the seedless fraction of the pomace differ from the seed fraction in terms of polysaccharide composition? Which fraction of berry pomace is the best source for obtaining functional berry polysaccharides?

## 2. Results and Discussion

### 2.1. Alcohol-Insoluble Solids Composition

According to data presented in Table 1, juices contained the lowest AIS (up to 50 mg/g), whereas raspberry and blackberry fruits contained over 300 mg/g. Pomace was characterized by AIS at the level of 630–840 mg/g. The main component of the AIS of fruits was galacturonic acid, then glucose derived from cellulose, then xylose, arabinose, galactose, and mannose. Mainly homogalacturonan and arabinogalactan passed into the juice. When we compare the content of AIS of juice, fruit, and pomace, we can easily see that only a small portion of polysaccharides passes into the juice. Most of the polysaccharides stay in the so-called waste pomace. Berry pomace is a valuable raw material for obtaining dietary fiber and ellagitannin preparations [11,13].

In the pomace, cellulose and xylans were densified. During the juice production, pectinases were used to hydrolyze pectin to facilitate the process of pressing. That is why galacturonic acid passes into the juice. The least soluble and non-hydrolysable cellulose and xylans were retained in the pomace. Similarly, in the raspberry pomace, especially in the seeds, 90% of the polyphenols, mostly tannins, are retained during the juice production [4]. Berry juice can be cloudy or clear. When juice is cloudy, it contains pectin, and its turbidity and viscosity, as well as nutrient retention, are higher. When pectinases are used to obtain clear juice, the process of pressing is easier, and the juice is clear, but part of the pectin, along with polyphenols, especially ellagitannins, is lost, and the juice turbidity and viscosity are much lower [25].

Studies have shown that by dividing berry pomace into seed and pulp (seedless) fractions, it is possible to obtain dietary fiber preparations with different polysaccharide compositions and different physical properties [11,13]. Dividing pomace into fractions is more economically justified for obtaining raw material with different compositions and properties than separating fruit into morphological parts [26], which is very important for scientific research but not so much for economic value. Pomace can be obtained in high quantities in the food industry as a waste. Pomace after juice production is not a homogeneous material. Sieving on appropriate sieves (with a mesh diameter of 1 mm for blackberries and raspberries) allows the division of berry pomace into pulp (seedless) and seed parts. Diversified in terms of protein, fat, and polyphenol content [11], they are a rich source of dietary fiber. Berry seeds have a tough outer coating that is not disrupted during the digestion processes in the gastrointestinal tract. When fine grinding is applied, only then can the full potential of seeds in the form of polysaccharides, protein, oil, and polyphenols be used [10,11]. Seedless fraction, on the other hand, consists of peels, cell walls, and fruit flesh. Physical differences are accompanied by differences in chemical composition. In our research, we concentrated only on the composition of the dietary fiber, and in the preparation steps (AIS procedure), the product was divided into sugars, polyphenols, and oils. The seed fraction was characterized by a higher content of xylans, and the seedless fraction contained equal amounts of arabinans, xylans, and galactans. He et al. [27] obtained the blackberry crude polysaccharides (BCP) composed of 95.44% glucose, 2.01% arabinose, 1.81% galactose, and 0.74% glucuronic acid as a result of extraction with 70% ethanol. These polysaccharides improved the quality of poultry breast meat. Yang et al. 2022 [18] isolated a homogeneous acidic polysaccharide (RPP-2a), with a weight-average molecular weight (MW) of 55 582 Da, from the pulp of raspberries through DEAE-Sepharose Fast Flow and Sephadex G-200 chromatography. The polysaccharide consisted of rhamnose, arabinose, galactose, glucose, xylose, galacturonic acid, and glucuronic acid, with a molar ratio of 15.4:9.6:7.6:3.2:9.1:54.3:0.8, respectively. Yu et al. [16] isolated a water-soluble polysaccharide named RCP-II from raspberry fruits, which was an acidic heteropolysaccharide mainly composed of galacturonic acid, rhamnose, arabinose, xylose, glucose, and galactose in a molar ratio of 1.00:0.55:1.19:0.52:0.44:1.90, respectively. RCP-II was a low-molecular-weight polysaccharide, and the average molecular weight was 4013 Da. The characteristic absorptive peaks of polysaccharide structure were determined by IR, and the presence of alpha-galactose and beta-arabinose residues was confirmed by NMR analysis. RCP-II was proven to have certain antioxidant capabilities and non-enzymatic glycation inhibition activity. The chemical composition of a polysaccharide may influence its biological properties. Based on their structural information and immune-enhancing activity data using an artificial neural network Lu et al. [17] found that next to molecular weight, the content of arabinose, galactose, or glucuronic acid, as well as glycosyl linkage patterns of →3)-Arap-(1→, Araf-(1→, →4)-Galp-(1→ have the strongest influence on immunomodulatory activities.

### 2.2. Water-Binding Capacity, Swelling, and Oil-Holding Capacity

Analyzing the obtained results, it can be stated that differences in chemical composition caused differences in physicochemical properties (Table 2). Raspberry fruit and blackberry fruit were characterized by 6.5 and 6.7 g water/g WBC values. Seedless fraction WBC values were higher than for the fruits, while the seeds fraction had WBC values significantly lower than the fruits or seedless fraction (raspberry—4.2 g water/g and blackberry—2.7 g water/g). The swelling of fruit fiber was 4.8 and 5.6 mL/g for raspberry and blackberry, respectively. Again, the seedless fraction expressed the highest swelling properties, 11.1 and 8.5 mL/g for the raspberry and blackberry seedless fractions, respectively, and the seed fraction the lowest, 3.8 and 3.3 mL/g for the raspberry and blackberry, respectively. The hydration properties of fibers depend on the chemical structure of the component polysaccharides, as well as factors such as porosity, particle size, ionic form, pH, temperature, ionic strength, and type of ions in solution [28]. The method of obtaining the polysaccharide may affect its properties. Rivas et al. [24] obtained polysaccharides from pomegranate peel conventionally and by the SFE method. The swelling and fat adsorption capacity values of the conventionally obtained polysaccharide were significantly higher than those found for SFE samples, while water retention capacity was lower for the conventionally obtained polysaccharide. Fruit and some cereal processing by-products had water holding capacities from 4.89 g/g (defatted rice bran) to 20.3 g/g for asparagus by-products, while swelling in water for sugar beet pulp was 11.5 and for algae 13.8 mL/g [28]. Rivas et al. [7] found that dietary fiber concentrates from winemaking by-products were characterized by 4.57–9.11 g/g water retention capacities. Tana et al. [8] found that while wheat bran had a WBC of 3.92 g/g konjac flour, pregelatinized waxy maize starch, guar gum, and xanthan gum were characterized by WBC over 20 g/g. They also stated that the physicochemical properties of dietary fiber may affect postprandial satiety in model research on male rats. WBC of dietary fiber preparations commercially available from wheat fiber Vitacel (WF 200, WF 400, WF 600), manufacturer J. Rettenmaier & SohneGmbH, Rosenberg, Germany, was 8.6, 11, and 4.9 g/g, respectively. The preparations were concentrated in dietary fiber (96%) and consisted mostly of cellulose (72%), then hemicellulose (25.5%), and lignin (0.5%). The fiber of the series Vitacel was found to have a number of positive functional and technological properties that allow recommending it for all kinds of meat, fish, and confectionery products [9]. Oil-holding capacities for fruit fiber were 16.5 and 12.2 g oil/g for raspberry and blackberry, respectively. Pomace had those values, 8.8 and 8.5 g oil/g, respectively. When pomace was divided into a seedless fraction and seed fraction, again the seedless fraction expressed higher values compared to the seed fraction (11.7 and 9.5 g oil/g vs. 8.0 and 9.0 g oil/g). Oil-holding capacities can be very diversified depending on fiber origin. Elleuch [28] found that OHC can vary from 1 for mango dietary fiber concentrate to 11.3 g oil/g for sugarcane bagasse (>0.3 mm), but high-pressure micronization creating 7.23 µm carrot insoluble fiber can increase the oil-holding capacities up to 56 g oil/g compared to 1.92 g oil/g for control carrot fiber (123 µm). Rivas et al. [7] found that dietary fiber concentrates from winemaking by-products were characterized by 3.76–5.48 g oil/g oil retention capacity. In the case of fat absorbability of dietary fiber preparations commercially available from wheat fiber Vitacel (WF 200, WF 400, WF 600), the values were from 3.7 to 12 g oil/g [9]. To sum up, berry pomace fiber could also be used as an ingredient in the bakery and meat industry, thanks to its relatively high water-binding capacity, swelling, and oil-holding capacity, with the seedless fraction being especially prominent.

### 2.3. Sequential Polysaccharide Extraction

Sequential extraction of polysaccharides allows the division of dietary fiber into polysaccharides with different properties. First stage: water releases weakly bound pectin (WSP—water-soluble pectin); second stage: CDTA releases pectin linked by calcium bonds (ChSP—chelating agent soluble pectin); third stage: 0.1 mol/L Na_2_CO_3_ releases highly methylated pectin and dissolves pectin linked by ester bonds (DASP—diluted alkali-soluble pectin); fourth stage: 4 mol/L NaOH + 1 g/L NaBH_4_ releases hemicelluloses and pectin with a high degree of branching strongly bound to cellulose microfibrils (CASP—concentrated alkali-soluble polysaccharides); fifth stage: washing with water extracts the remaining branched pectin (WR—water residue). What remains (residue fraction) is insoluble and consists mainly of cellulose [29]. While polysaccharides on an industrial scale are usually obtained by hot water extraction [19], the additional step between water extraction and weak alkali extraction allows for the extraction of pectin that needs calcium ions for binding, increasing the yield. The pectin obtained by water extraction has a higher degree of methylation than pectin obtained by chelating agent extraction [29], which influences the gelling abilities of the polysaccharide. The lower the degree of methylation, the more calcium ions are required, but less sugar.

As a result of the sequential extraction of polysaccharides, it was shown (Table 3) that raspberry fruit fiber, in addition to the residue fraction, was also rich in DASP and ChSP fractions. The fiber obtained from pomace in its entirety was characterized by a much lower share of these two fractions. The cellulose Residue fraction was thickened, and the pectin fractions, i.e., WSP, ChSP, and DASP, were lost. The share of the CASP fraction in pomace fiber and fruit fiber was similar (17% AIS vs. 18%). When the pomace was divided into seedless and seed fractions, the share of Residue in the seedless fraction decreased (22% AIS vs. 60% in seeds), and the share of the hemicellulose fraction increased 24% vs. 11% in seeds. In the case of the seedless fraction, the share of pectin fractions WSP, ChSP, DASP, and even WR was higher.

Blackberry fruit fiber (Table 4) was not characterized by such a significant share of ChSP and DASP fractions as raspberry fruit fiber. The main fraction here was the Residue fraction (50% AIS), followed by a large share of the CASP hemicellulose fraction (13% AIS). Blackberry pomace as a whole was characterized by a concentrated Residue fraction (up to 58% AIS) in relation to fruit fiber. The seedless fraction was again poorer in the Residue fraction (26% AIS vs. 50%) than the seedless fraction and richer in the CASP hemicellulose fraction (25% vs. 5% AIS in seeds) and the weak alkali-soluble pectin fraction DASP (13 vs. 6% AIS), ChSP (10 vs. 7% AIS), and, to a lesser extent, in the WSP fraction (3 vs. 2.8%) and WR fractions (3.5 vs. 2.5%).

As a result of the sequential extraction of polysaccharides in the case of berry fruit fiber, the main fraction was the Residue fraction, followed by the weak alkali-extractable pectin fraction, i.e., the DASP fraction, and the hemicellulose fraction, i.e., polysaccharides that require strong alkali for extraction, i.e., the CASP fraction. Similar shares were found for water-extractable pectin, i.e., WSP fractions (8–9%), and chelating agent extractable pectin, i.e., ChSP fractions (8–9% AIS). Berry fruit pomace fiber contained mainly the Residue fraction (40% AIS), followed by CASP (16% AIS), DASP and ChSP (6–7% AIS), and WSP and WR (3% AIS). In the seed part, the Residue fraction had a much higher share than in the seedless part (52–57% AIS vs. 24–36% AIS), while in the seedless part, the share of CASP hemicelluloses was increased (24–28% AIS vs. 12–15% in seeds). In the seedless part, there was also more water-soluble pectin, i.e., the WSP fraction (4–5% vs. 2–3% in seeds).

In the next stage, the chemical composition of the fractions obtained by sequential extraction using the GC-FID method after hydrolysis in 1 mol/L sulfuric acid and conversion of the obtained simple sugars into volatile acetate alditols was analyzed, and galacturonic acid was determined using the spectrophotometric method. The main neutral sugar of dietary fiber in raspberry and blackberry fruits was glucose derived from cellulose (mainly the Residue fraction), followed by xylose, arabinose, galactose, and mannose. Compared to fiber from fruits, there was more cellulose and xylans in the pomace. Hotchkiss et al. [30] also found that the strawberry pomace contained mostly glucose and xylose. In our research, the first three obtained fractions, i.e., WSP, ChSP, and DASP, contained most of the galacturonic acid in all cases. While the CASP, WR, and Residue fractions contained only a few percent of galacturonic acid each. The fruits contained more galacturonic acid than the pomace, except for raspberries, where the amount of galacturonic acid in fruits and pomace was similar. After dividing the pomace into the seedless and seed parts, it turned out that the seedless fractions contained a similar profile of galacturonic acid as the fruits, while the seeds contained significantly lower shares of galacturonic acid.

## 3. Materials and Methods

### 3.1. Plant Material and Method of Preparing the Material for Further Analysis

A sample of berries of 1 kg each (raspberry (*Rubus idaeus* L.) and blackberry (*Rubus fruticosus* L.) were subjected to laboratory processing into juice under conditions imitating industrial conditions. Frozen raspberries and frozen wild blackberries were obtained from Cajdex (Lodz, Poland). The fruit was ground (Zelmer, Rzeszów, Poland). Enzymation of the fruit pulp was carried out for 1 h at 45 °C using the enzyme preparation Rohapect 10 L at a dose of 0.2 mL/kg of fruit. Then the juice was pressed on a manual laboratory press. Reflecting industrial conditions, water flushing (100% of the pomace weight) for 0.5 h was used, and the mass was pressed for the second time. The efficiency of obtaining juice was 80%, and the efficiency of obtaining wet pomace was 8%, with a water content of 30%. After freeze-drying at −32 °C for 48 h in the Christ Alpha 1–2 Plus freeze dryer (Martin Christ Gefriertrocknungsanlagen GmbH, Osterode am Harz, Germany), the obtained pomace was sieved through 1 mm sieves. Dried raspberry and blackberry pomace constituted 75% seeds and 25% pulp (seedless fraction). Sublimation-dried fruits, whole pomace, and pomace divided into seed and seedless fractions after grinding in liquid nitrogen in an analytical mill (IKA, Staufen, Germany, were subjected to the procedure of obtaining AIS (alcohol insoluble solids, Section 3.2) as described in [29]. The juices were precipitated in 78% ethyl alcohol and were also used to obtain AIS. In the obtained AIS, the composition of cell wall polysaccharides was determined using the derivatization method for volatile alditols and GC-FID determination (Section 3.4, Section 3.5 and Section 3.6), galacturonic acid content by the spectrophotometric method (Section 3.7), and physicochemical properties in the form of WBC (water-binding capacity, Section 3.8) according to [31]. In the next stage, sequential extraction of pectin from AIS obtained from freeze-dried fruits, whole pomace, and pomace divided into seed and seedless fractions was carried out (Section 3.3). The chemical composition of the fractions obtained as a result of sequential extraction was determined by GC-FID after hydrolysis in 1 mol/L sulfuric acid and conversion of the obtained simple sugars into volatile acetate alditols (Section 3.4, Section 3.5 and Section 3.6), and galacturonic acid was determined by spectrophotometry (Section 3.7).

### 3.2. Alcohol-Insoluble Solid (AIS) and Sequential Polysaccharide Extraction

**Figure 1:**
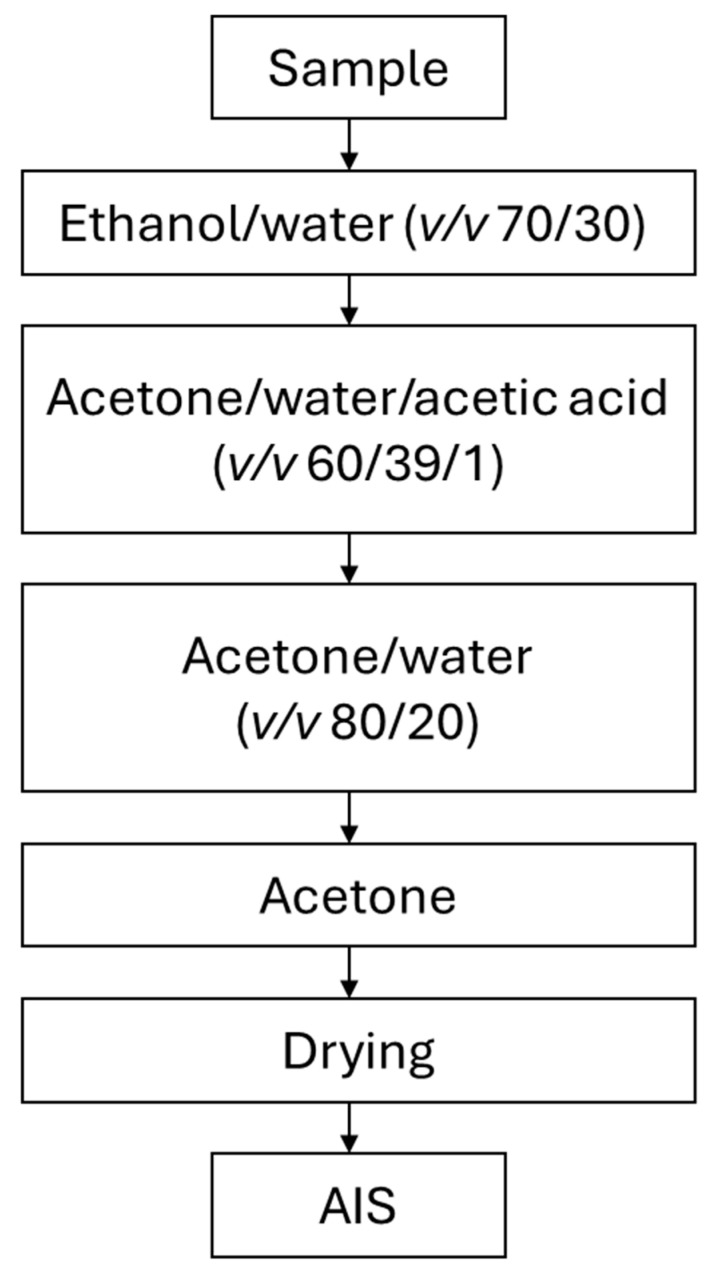
Alcohol-insoluble solids procedure.

AIS was obtained according to [32] (Figure 1). Approximately 5 g of dried powdered sample was mixed with 50 mL of 70% ethanol for 1 h at room temperature. Then, the sample was filtered under a vacuum, the residue was washed several times with 70% ethanol. Then, the sample was washed twice with 25 mL of acetone/water/acetic acid mixture (*v*/*v*/*v* 60/39/1), twice with acetone/water mixture (*v*/*v* 80/20), and twice with 100% acetone. The resulting solids were dried at 40 °C and weighed. The procedure was performed in duplicate for each sample material.

**Figure 2:**
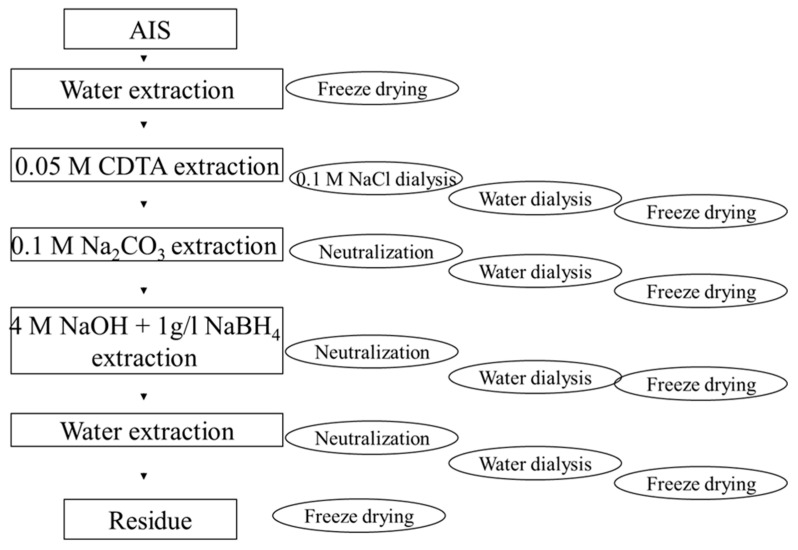
Sequential pectin extraction scheme.

Sequential polysaccharide extraction was performed according to [29] (Figure 2). AIS samples (1.5 g) were extracted three times with 45 mL of water (2 h, 25 °C) in tubes, and the solids were separated by centrifugation (10,000× *g*) from the supernatant. The supernatants were combined and freeze-dried in a Christ Alpha 1-2 Plus freeze-dryer (Osterode am Harz, Germany), giving a Water-Soluble Pectin fraction (WSP). Then, the solid residue was extracted with 45 mL of 0.05 mol/L CDTA (trans-1,2-diaminocyclohexane-N,N,N′,N′-tetraacetic acid) at pH 5 for 16 h and then twice for 4 h, all at 25 °C. The solids were separated by centrifugation, supernatants were combined and dialyzed in dialysis tubing MWCO 12,400 (Sigma-Aldrich, Poznań, Poland) against 0.1 mol/L NaCl solution and then against water to 0 conductivity, giving Chelating Agent Soluble Pectin fraction (ChSP). Then, the solid residue was extracted with 0.1 mol/L Na_2_CO_3_ for 16 h and again for 6 h, all at 25 °C. The obtained supernatants were combined and neutralized with acetic acid to pH 4–5 and then dialyzed against water and freeze-dried, giving Diluted Alkali-Soluble Pectin fraction (DASP). Then, the residue was extracted with 4 mol/L NaOH + 1 g/L NaBH_4_ for 16 h and again for 8 h, all at 20 °C. The supernatants were combined and neutralized with acetic acid to pH ~ 4, then dialyzed against water and freeze-dried, giving Concentrated Alkali-Soluble Polysaccharides (CASP). Then the residue was extracted with 45 mL portions of water until pH 7. All the supernatants were combined, neutralized to pH 4–5, dialyzed against water, and freeze-dried, giving the Water residue fraction (WR). The solid residue after extractions was also freeze-dried, giving the Residue fraction.

### 3.3. Cell Wall Hydrolysis

Hydrolysis was carried out according to [29]. Approximately 8–12 mg of sample (AIS and Residue fraction) were weighed into test tubes, then 250 µL of 72% H_2_SO_4_ solution was added and mixed. The samples were incubated for 1 h at room temperature (prehydrolysis), then 1 mL of inositol solution with a concentration of 1 mg/mL (internal standard for GC), and 1.7 mL of distilled water was added. The samples were incubated at 100 °C for 3 h and then cooled to room temperature.

### 3.4. Pectin Hydrolysis

Approximately 8–12 mg of sample (AIS, sequential polysaccharide extraction fractions) were weighed into test tubes, 1 mL of inositol solution (internal GC standard) at a concentration of 1 mg/mL was added, and mixed. Then 1 mL of 2 mol/L H_2_SO_4_ solution was added, and the contents were mixed. The samples were heated at 100 °C for 3 h. The subsequent hydrolysis procedure was the same as the procedure described in the cell wall hydrolysis. The procedure did not allow total cellulose hydrolysis into glucose but only hydrolyzed pectin and hemicelluloses, giving NGlc—non-cellulosic glucose. All in triplicate. Cellulosic glucose was calculated by subtracting NGlc from total Glc.

### 3.5. Derivatization and Gas Chromatography

Here, 1 mL of each sample (obtained as described in Section 3.4 and Section 3.5) was transferred into different tubes and neutralized with concentrated NH_3_·H_2_O to make the solution pH greater than 9. Then, 0.1 mL of NaBH_4_ solution (100 mg/mL solution prepared in 3 mol/L NH_3_·H_2_O) was added and incubated for one hour at room temperature. After incubation, 0.1 mL of pure acetic acid was added to the samples. 1 mL of the prepared solution was transferred to a glass test tube and then added in the following order: 0.2 mL of N-methyl-imidazole, 3 mL of acetic anhydride, 5 mL of cold distilled water, and 3 mL of CH_2_Cl_2_. The resulting aqueous phase was removed by the water pump, and the organic phase was washed four times with 5 mL of 0.5 mol/L KHCO_3_ solution. The solutions remaining after washing were dissolved in 0.5 mL of CH_2_Cl_2_ and analyzed by GC. The procedure with prehydrolysis allowed a total of cellulose hydrolysis into glucose. All in triplicate (the procedure was performed in triplicate for each tested material).

**Figure 3:**
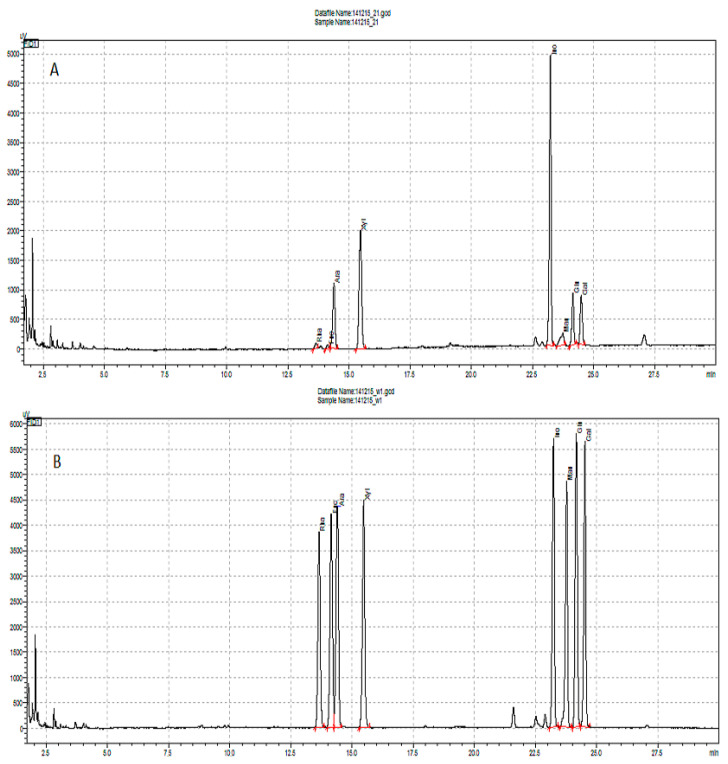
Raspberry fruit after pectin hydrolysis procedure, GC-FID chromatograph (**A**), and standards chromatograph (**B**). At 13.640 min rhamnitol per acetyl; 14.131 min fucitol per acetyl; 14.377 min arabinitol per acetyl; 15.461 min xylitol per acetyl; 23.225 min inositol per acetyl; 23.786 min mannitol per acetyl; 24.146 min glucitol per acetyl; 24.492 min galactitol per acetyl.

The analysis was performed using the Shimadzu GC-2010 Plus chromatograph (Tokyo, Japan). The GC was equipped with the AOC-20i autosampler and Hydrogen Peak Scientific hydrogen generator (Inchinnan, Scotland, UK). Hydrogen, helium, and air were used for the separation. The Zebron ZB-5 column (Phenomenex, Torrance, CA, USA) with dimensions of 30 m × 0.25 mm × 0.25 μm was used. Detection was carried out using a flame ionization detector (FID). The gas flow rate for the FID detector was 30 mL/min for helium, 40 mL/min for hydrogen, and 400 mL/min for air, respectively. The temperature gradient was used as follows: 15 min to 170 °C, then 200 °C at 6 °C/min, then 10 min at 200 °C, and cooling to the initial temperature for 10 min. The injection temperature was 250 °C, and the volume was 1 μL in split mode (ratio 1:25). The sample flow rate through the column was 1.53 mL/min (total flow of 42.8 mL/min, purge flow 3.0 mL/min). Identification of the sugar alditols contained in the samples was made on the basis of the peak retention times on the chromatograms of the samples with the chromatograms of the standard solutions (rhamnose, fucose, arabinose, xylose, mannose, galactose, glucose, all Sigma-Aldrich). Inositol was used as an internal standard (Sigma-Aldrich). Figure 3 presents a chromatogram of raspberry fruit AIS after pectin hydrolysis.

### 3.6. Galacturonic Acid

The analysis was carried out according to [29]. Under the conditions of the assay, complete hydrolysis of pectic substances to galacturonic acid occurs. Galacturonic acid reacts with sulfuric acid to form 5-formyl-2-furancarboxylic acid. In turn, the derivative reacts with m-hydroxydiphenyl (MHDP) to form pink-colored complexes with maximum absorbance at 520 nm. In tubes, 0.5 mL of the sample (obtained by the procedure of cell wall hydrolysis or by solubilizing the pectin fraction) was mixed with 3 mL of 0.0125 mol/L sodium tetraborate (Na_2_B_4_O_7_) reagent in concentrated sulfuric acid H_2_SO_4_ (96%) and carefully mixed. The tubes were incubated at 80 °C for 20 min. Then, the reaction was stopped by placing the tubes in an ice-water bath until the room temperature was reached. Then 50 μL of MHDP solution was added, the contents of the tubes were mixed, and absorbance was measured at a wavelength of 520 nm exactly after 10 min (at the spectrum maximum). Galacturonic acid was used as a standard (Sigma-Aldrich). All in triplicate.

### 3.7. Water-Binding Capacity

The hydration properties of fibers were carried out according to [29]. 100 mg of AIS was weighed in test tubes with 25 mL of distilled water at 4 °C. The tubes were centrifuged for 20 min at 10,700× *g* at 20 °C, the supernatant was removed immediately, and the pellet was dried at room temperature on a Schott funnel n° 1 for 2 h. The wet sediment was weighed, then dried at 120 °C for 2 h and weighed. WBC was expressed as the amount of water/dry sample. All in triplicate.

### 3.8. Swelling

Here, 100 mg of AIS was weighed in test tubes with a scale, and 5 mL of distilled water at 20 °C was added. The tubes were carefully mixed and stored for 24 h. Swelling is calculated as milliliters of swollen material (swollen material column)/output weight of the product [29]. All in triplicate.

### 3.9. Oil-Holding Capacity

Here, 1 g of AIS was weighed in test tubes with 5 mL of oil at room temperature. Then, stored for 24 h. The tubes were then centrifuged for 20 min at 10,700× *g* at 20 °C; the supernatant was removed immediately. The sediment was weighed. OHC was expressed as oil quantity/dry sample (g/g) [9]. All in triplicate.

### 3.10. Statistical Analysis

The results are expressed as means and standard deviations. One-way ANOVA with Tukey test, a statistical significance of *p* ≤ 0.05, was used (Statistica 12, Statsoft, Kraków, Poland).

## 4. Conclusions

Most of the fruit polysaccharides stay in the pomace, as only part of the soluble polysaccharides pass to the juice. Berry pomace, being a waste of the food industry, can be used as a source of raw material for obtaining dietary fiber preparation and the extraction of polysaccharides.

When berry pomace is divided into pulp (seedless) and seed parts, it is possible to obtain fruit fiber with different chemical compositions and properties. The seedless part is similar to the fruit, where the flesh is a dominant part, not the seeds, with more hemicelluloses and homogalacturonan than the pomace seed part, which contains more cellulose. The differences in chemical composition caused differences in physical properties. Fruit as well as the seedless fraction express higher WBC, swelling, and oil-holding capacities than the seed fraction. However, all fruit fibers had rather high physical properties (water-binding capacities were from 2.7 to 14.9 g/g, swelling from 3.3 to 11.1 mL/g, and oil-holding capacities from 8.0 to 16.5 g/g) and could be used in the food industry as texturizing agents.

The sequential extraction of polysaccharides of berry fruit fiber showed that the main fraction was the Residue fraction, then the weak alkali extractable pectin fraction (DASP), and the hemicellulose fraction (CASP). Water-extractable pectin (WSP) and chelating agent extractable pectin (ChSP) both constituted 8–9% of AIS. In the case of pomace, the main fraction was the Residue fraction (40% AIS), followed by CASP (16% AIS), DASP and ChSP (6–7% AIS), and WSP and WR (3% AIS). In the seed part, the Residue fraction had a much higher share than in the seedless part (52–57% AIS vs. 24–36% AIS), while in the seedless part, the share of CASP hemicelluloses was increased (24–28% AIS vs. 12–15% in seeds). In the seedless part, there was also more water-soluble pectin (WSP) (4–5% vs. 2–3% in seeds).

Separating berry pomace into seeds and seedless fractions allows for the obtaining of different functional products. Seedless fraction is rich in hemicellulose and has higher water-binding properties and oil-holding capacities, which is why it has a higher potential for digestion regulation by favorable gut microbiota modulation and increased short-chain fatty acid production. Seed fractions rich in cellulose could be used in the food industry, especially in confectionery and bakery. Separating berry pomace into seedless and seed fractions also allows the extraction of polysaccharides with different chemical compositions and properties, with the seedless fraction being an excellent source of functional berry polysaccharides.

To further expand the knowledge on the properties of berry polysaccharides from seeds and seedless fractions, studies on the digestion characteristics, short-chain fatty acid production, blood lipid parameters, and impacts on gut microbiota using animal models are planned.

## Figures and Tables

**Table 1:** Yields (mg/g) and sugar composition (mg/g AIS) of the alcohol-insoluble solids.

	Yield of AIS [mg/g]	Rha [mg/g]	Fuc [mg/g]	Ara [mg/g]	Xyl [mg/g]	Man [mg/g]	Gal [mg/g]	Noncell Glc [mg/g]	Cell Glc [mg/g]	GalA [mg/g]
Raspberry										
Fruit	394 ± 4 f	1 ± 0 c	1 ± 0 cd	16 ± 5 c	39 ± 9 c	6 ± 1 b	12 ± 4 d	12 ± 0 c	64 ± 6 b	305 ± 21 b
Pomace total	812 ± 10 a	1 ± 0 c	1 ± 0 cd	9 ± 2 e	52 ± 10 ab	6 ± 3 b	9 ± 1 e	5 ± 2 d	66 ± 13 b	219 ± 52 c
Pomace seedless fraction	717 ± 1 d	1 ± 0 d	2 ± 1 b	11 ± 2 de	35 ± 10 c	8 ± 5 ab	18 ± 3 cd	12 ± 2 c	90 ± 29 ab	177 ± 32 e
Pomace seed fraction	775 ± 11 ab	1 ± 0 cd	1 ± 0 cd	9 ± 2 e	54 ± 19 ab	4 ± 1 c	7 ± 2 e	3 ± 0 d	49 ± 3 b	172 ± 14 e
Juice	38 ± 0 h	9 ± 1 a	ND	34 ± 2 b	15 ± 1 e	7 ± 2 b	41 ± 2 a	20 ± 8 b	ND	278 ± 16 bc
Blackberry										
Fruit	333 ± 11 fg	1 ± 0 c	1 ± 0 cd	20 ± 3 c	46 ± 14 ab	5 ± 1 bc	10 ± 1 de	3 ± 0 d	59 ± 17 b	247 ± 20 bc
Pomace total	731 ± 24 cd	1 ± 0 cd	1 ± 0 c	15 ± 3 c	47 ± 9 ab	5 ± 2 bc	9 ± 2 e	4 ± 1 d	72 ± 8 ab	176 ± 22 e
Pomace seedless fraction	631 ± 7 e	1 ± 0 cd	3 ± 0 a	31 ± 4 b	25 ± 9 d	8 ± 5 ab	16 ± 5 cd	12 ± 7 c	102 ± 12 a	236 ± 30 bc
Pomace seed fraction	766 ± 2 bc	1 ± 0 c	ND	8 ± 2 e	58 ± 18 a	4 ± 2 c	5 ± 2 e	2 ± 0 d	62 ± 6 b	184 ± 48 e
Juice	49 ± 0 h	6 ± 0 b	ND	67 ± 2 a	3 ± 1 f	10 ± 2 ab	27 ± 2 b	47 ± 9 a	ND	363 ± 59 a
*p*	0.000	0.000	0.000	0.000	0.000	0.001	0.000	0.000	0.000	0.000

Within the same column, means with different letters are significantly different at *p* ≤ 0.05. ND—not detected. Yield of AIS—yield of AIS on dry weight basis, Rha—rhamnose, Fuc—fucose, Ara—arabinose, Xyl—xylose, Man—mannose, Gal—galactose, GalA—galacturonic acid, Noncell Glc—glucose determined without cellulose hydrolysis, Cell Glc—glucose exclusively from cellulose, calculated as glucose determined in hydrolysis minus glucose determined without hydrolysis of cellulose.

**Table 2:** Water-binding capacity, swelling, and oil-holding capacity of fibers (AIS).

	Water-Binding Capacity [g Water/g]	Swelling [ml/g]	Oil-Holding Capacity [g Oil/g]
Raspberry			
Fruit	6.5 ± 0.9 c	4.8 ± 0.2 d	16.5 ± 0.3 a
Pomace total	8.5 ± 0.2 b	3.7 ± 0.0 ef	8.8 ± 0.1 ef
Pomace seedless fraction	14.9 ± 0.4 a	11.1 ± 0.1 a	11.7 ± 0.1 c
Pomace seed fraction	4.2 ± 1.1 d	3.8 ± 0.2 e	8.0 ± 0.0 g
Blackberry			
Fruit	6.7 ± 0.9 bc	5.6 ± 0.2 c	12.2 ± 0.0 b
Pomace total	4.5 ± 1.0 d	3.8 ± 0.0 e	8.5 ± 0.1 f
Pomace seedless fraction	13.0 ± 1.7 a	8.5 ± 0.3 b	9.5 ± 0.1 d
Pomace seed fraction	2.7 ± 0.3 d	3.3 ± 0.1 f	9.0 ± 0.0 e
*p*	0.000	0.000	0.000

Within the same column, means with different letters are significantly different at *p* ≤ 0.05.

**Table 3:** Sequential extraction of polysaccharides of raspberry fruit and pomace: yield and sugar composition of extracts (mg/g).

		Yield [mg/g]	Rha [mg/g]	Fuc [mg/g]	Ara [mg/g]	Xyl [mg/g]	Man [mg/g]	Gal [mg/g]	Glc [mg/g]	GalA [mg/g]
Raspberry fruit	WSP	74	2 ± 0 c	1 ± 0 d	47 ± 5 a	24 ± 2 h	15 ± 1 c	25 ± 2 cd	6 ± 2 fc	436 ± 38 a
	ChSP	220	1 ± 0 d	ND	10 ± 3 f	5 ± 2 i	6 ± 5 d	3 ± 1 h	2 ± 4 gf	177 ± 0 ef
	DASP	224	1 ± 0 d	ND	18 ± 5 d	6 ± 0 i	2 ± 1 f	6 ± 1 h	3 ± 0 gg	111 ± 24 fgh
	CASP	182	2 ± 0 c	3 ± 0 c	26 ± 1 d	59 ± 4 f	8 ± 0 d	17 ± 1 e	52 ± 3 ef	7 ± 0 i
	WR	58	2 ± 0 c	ND	8 ± 0 f	59 ± 7 f	1 ± 1 g	4 ± 0 h	12 ± 2 f	27 ± 2 i
	Residue	304	1 ± 0 d	ND	3 ± 0 g	118 ± 2 b	6 ± 1 d	5 ± 0 h	253 ± 3 c	67 ± 5 ghi
Raspberry pomace total	WSP	57	1 ± 0 d	1 ± 0 d	20 ± 3 d	34 ± 5 g	8 ± 2 d	18 ± 3 e	16 ± 2 f	317 ± 0 bc
	ChSP	49	1 ± 0 d	ND	7 ± 1 f	4 ± 0 i	3 ± 3 f	1 ± 1 i	2 ± 1 g	150 ± 23 f
	DASP	83	1 ± 0 d	ND	18 ± 1 d	7 ± 1 i	1 ± 1 g	7 ± 1 h	6 ± 0 f	222 ± 115 de
	CASP	168	2 ± 0 c	5 ± 0 b	31 ± 1 c	88 ± 2 e	22 ± 0 b	28 ± 1 b	64 ± 13 e	68 ± 0 ghi
	WR	56	2 ± 0 c	ND	13 ± 2 e	52 ± 4 f	1 ± 0 g	4 ± 1 h	13 ± 0 f	33 ± 2 hi
	Residue	463	1 ± 0 d	ND	6 ± 0 g	104 ± 10 cd	5 ± 1 e	5 ± 0 h	345 ± 25 b	63 ± 6 ghi
Raspberry pomace seedless	WSP	64	1 ± 0 d	2 ± 0 d	18 ± 2 d	40 ± 4 g	8 ± 1 d	23 ± 2 d	18 ± 1 f	252 ± 18 cd
	ChSP	70	1 ± 1 d	ND	15 ± 4 d	6 ± 2 i	2 ± 2 f	5 ± 2 h	0 ± 1 g	335 ± 150 b
	DASP	99	1 ± 0 d	ND	19 ± 2 d	4 ± 0 i	1 ± 0 g	8 ± 0 g	3 ± 0 g	422 ± 20 a
	CASP	243	1 ± 0 d	7 ± 0 a	16 ± 0 d	106 ± 1 c	38 ± 0 a	41 ± 1 a	103 ± 1 d	65 ± 10 ghi
	WR	39	3 ± 1 bc	1 ± 0 d	19 ± 6 d	61 ± 3 f	2 ± 1 f	11 ± 4 f	51 ± 8 e	13 ± 7 i
	Residue	223	1 ± 0 d	ND	5 ± 0 g	30 ± 5 gh	5 ± 0 e	4 ± 0 h	605 ± 14 a	43 ± 5 hi
Raspberry pomace seed	WSP	13	1 ± 0 d	1 ± 0 d	13 ± 0 e	23 ± 3 h	9 ± 1 d	11 ± 1 f	17 ± 1 f	263 ± 10 cd
	ChSP	58	0 ± 1 d	1 ± 0 d	2 ± 1 g	4 ± 2 i	5 ± 1 e	1 ± 1 i	ND	122 ± 1 fg
	DASP	69	1 ± 0 d	ND	12 ± 3 e	6 ± 1 i	1 ± 1 g	5 ± 1 h	5 ± 1 f	378 ± 0 ab
	CASP	110	4 ± 0 b	4 ± 0 c	47 ± 2 a	97 ± 1 de	16 ± 1 c	27 ± 1 bc	48 ± 7 e	52 ± 3 ghi
	WR	30	6 ± 3 a	0 ± 1 d	37 ± 6 b	145 ± 21 a	3 ± 0 f	11 ± 4 f	9 ± 1 f	78 ± 5 ghi
	Residue	601	1 ± 0 d	ND	3 ± 0 g	111 ± 6 bc	6 ± 1 d	4 ± 0 h	246 ± 10 c	44 ± 10 ghi
	***p***		0.000	0.000	0.000	0.000	0.000	0.000	0.000	0.000

Within the same column, means with different letters are significantly different at *p* ≤ 0.05. ND—not detected. Rha—rhamnose, Fuc—fucose, Ara—arabinose, Xyl—xylose, Man—mannose, Gal—galactose, GalA—galacturonic acid, Glc—glucose, WSP—water-soluble pectin, ChSP—chelating agent soluble pectin, DASP—diluted alkali-soluble pectin, CASP—concentrated alkali-soluble polysaccharides, Water residue—WR, Residue—the remaining insoluble fraction.

**Table 4:** Sequential extraction of polysaccharides of blackberry fruit and pomace: yield and sugar composition of extracts (mg/g).

		Yield [mg/g]	Rha [mg/g]	Fuc [mg/g]	Ara [mg/g]	Xyl [mg/g]	Man [mg/g]	Gal [mg/g]	Glc [mg/g]	GalA [mg/g]
Blackberry fruit	WSP	50	3 ± 1 cde	1 ± 0 efgh	67 ± 2 abcd	10 ± 1 ijk	5 ± 1 fg	28 ± 2 bc	18 ± 0 ef	585 ± 50 c
	ChSP	82	1 ± 0 def	ND	24 ± 8 hijk	4 ± 1 k	ND	6 ± 2 ghij	ND	247 ± 39 efg
	DASP	67	4 ± 0 cd	ND	73 ± 10 abc	5 ± 0 k	2 ± 0 jk	15 ± 3 de	5 ± 1 f	465 ± 27 cd
	CASP	131	2 ± 0 def	6 ± 0 e	46 ± 1 efg	73 ± 2 de	18 ± 1 c	26 ± 1 c	73 ± 3 cde	50 ± 12 hi
	WR	44	8 ± 2 ab	ND	44 ± 7 efg	24 ± 3 i	1 ± 1 jk	12 ± 2 defg	3 ± 0 f	12 ± 0 i
	Residue	502	1 ± 0 def	ND	4 ± 0 kl	107 ± 8 ab	4 ± 0 fghij	4 ± 1 ij	266 ± 15 b	64 ± 7 hi
Blackberry pomace total	WSP	23	1 ± 0 ef	1 ± 0 e	26 ± 0 ghij	25 ± 0 ih	9 ± 1 de	15 ± 0 de	23 ± 1 def	355 ± 41 de
	ChSP	60	1 ± 0 ef	ND	10 ± 2 jkl	5 ± 1 k	2 ± 1 ghijk	2 ± 0 j	ND	240 ± 78 efg
	DASP	85	1 ± 1 def	ND	12 ± 5 ijkl	3 ± 1 k	1 ± 0 jk	4 ± 2 hij	6 ± 2 f	154 ± 5 fgh
	CASP	114	3 ± 0 cd	7 ± 0 b	57 ± 1 bcde	92 ± 1 bc	25 ± 1 b	33 ± 1 ab	80 ± 14 cd	45 ± 13 hi
	WR	44	6 ± 2 b	ND	32 ± 1 fghi	21 ± 2 ij	1 ± 0 jk	17 ± 2 d	15 ± 1 ef	15 ± 3 i
	Residue	577	1 ± 0 def	ND	4 ± 0 kl	115 ± 5 a	4 ± 0 fghij	4 ± 0 hij	277 ± 3 b	60 ± 3 hi
Blackberry pomace seedless	WSP	30	1 ± 0 def	3 ± 0 d	70 ± 12 abc	49 ± 7 fg	10 ± 3 e	30 ± 7 bc	14 ± 2 f	793 ± 154 b
	ChSP	102	3 ± 1 cdef	ND	76 ± 20 ab	5 ± 2 jk	2 ± 1 ghijk	13 ± 4 def	1 ± 0 f	264 ± 2 ef
	DASP	128	2 ± 1 def	ND	48 ± 8 def	3 ± 0 k	2 ± 1 jk	11 ± 2 defg	7 ± 2 f	927 ± 0 a
	CASP	248	2 ± 0 def	9 ± 0 a	37 ± 1 efgh	93 ± 5 bc	40 ± 1 a	38 ± 2 a	110 ± 5 c	42 ± 19 hi
	WR	35	9 ± 1 a	1 ± 0 efg	81 ± 9 a	23 ± 3 i	2 ± 0 ijk	32 ± 3 abc	18 ± 2 ef	11 ± 5 i
	Residue	257	1 ± 0 def	ND	13 ± 2 ijkl	40 ± 19 gh	6 ± 1 ef	6 ± 1 fghij	612 ± 87 a	47 ± 4 hi
Blackberry pomace seed	WSP	28	1 ± 0 def	1 ± 0 ef	19 ± 2 hijkl	17 ± 1 ijk	12 ± 2 e	9 ± 0 efghi	26 ± 2 def	241 ± 6 efg
	ChSP	65	ND	ND	3 ± 0 l	5 ± 1 k	1 ± 1 ijk	1 ± 0 j	ND	126 ± 6 ghi
	DASP	60	ND	ND	3 ± 0 l	3 ± 0 k	1 ± 0 jk	1 ± 0 j	4 ± 1 f	139 ± 63 fghi
	CASP	49	4 ± 0 cd	3 ± 0 d	55 ± 1 cde	62 ± 2 ef	5 ± 0 fgh	16 ± 0 d	28 ± 7 def	62 ± 4 hi
	WR	25	5 ± 2 bc	ND	30 ± 5 fghi	81 ± 5 cd	2 ± 0 ijk	11 ± 2 defgh	8 ± 1 f	18 ± 13 i
	Residue	503	1 ± 0 def	ND	2 ± 0 l	122 ± 3 a	5 ± 0 fghi	3 ± 0 ij	263 ± 6 b	45 ± 4 hi
	***p***	0.000	0.000	0.000	0.000	0.000	0.000	0.000	0.000	0.000

Within the same column, means with different letters are significantly different at *p* ≤ 0.05. ND—not detected. Rha—rhamnose, Fuc—fucose, Ara—arabinose, Xyl—xylose, Man—mannose, Gal—galactose, GalA—galacturonic acid, Glc—glucose, WSP—water-soluble pectin, ChSP—chelating agent soluble pectin, DASP—diluted alkali-soluble pectin, CASP—concentrated alkali-soluble polysaccharides, Water residue—WR, Residue—the remaining insoluble fraction.

## Data Availability

The data presented in this study are available on request from the corresponding author.

## Abbreviations

The following abbreviations are used in this manuscript:

Ara	Arabinose
CASP	Concentrated alkali soluble polysaccharides
CDTA	Trans-1,2-diaminocyclohexane-N,N,N′,N′-tetraacetic acid
ChSP	Chelating agent soluble pectin
DASP	Diluted alkali-soluble pectin
Fuc	Fucose
Gal	Galactose
GalA	Galacturonic acid
Glc	Glucose
Man	Mannose
MHDP	m-hydroxydiphenyl
OHC	Oil-holding capacity
Rha	Rhamnose
WBC	Water-binding capacity
WR	Water residue
WSP	Water soluble pectin
Xyl	Xylose
